# Three Weeks of Murine Hindlimb Unloading Induces Shifts from B to T and from Th to Tc Splenic Lymphocytes in Absence of Stress and Differentially Reduces Cell-Specific Mitogenic Responses

**DOI:** 10.1371/journal.pone.0092664

**Published:** 2014-03-24

**Authors:** Fanny Gaignier, Véronique Schenten, Marcelo De Carvalho Bittencourt, Guillemette Gauquelin-Koch, Jean-Pol Frippiat, Christine Legrand-Frossi

**Affiliations:** 1 Stress Immunity Pathogens Laboratory, EA7300, Lorraine University, Vandœuvre-lès-Nancy, France; 2 Research Center for Automatic Control of Nancy, UMR7039, Lorraine University, Vandœuvre-lès-Nancy, France; 3 French National Space Agency, Paris, France; Uniform Services University of the Health Sciences, United States of America

## Abstract

Extended space missions are known to induce stress and immune dysregulation. Hindlimb unloading is a ground-based model used to reproduce most spaceflight conditions. The aim of this study was to better characterize the consequences of prolonged exposure to hindlimb unloading on murine splenic lymphocyte sub-populations. To ensure that the observed changes were not due to tail restraint but to the antiorthostatic position, three groups of mice were used: control (C), orthostatic restrained (R) and hindlimb unloaded (HU). After 21 days of exposure, no difference in serum corticosterone levels nor in thymus and spleen weights were observed between HU mice and their counterparts, revealing a low state of stress. Interestingly, flow cytometric analyses showed that B cells were drastically reduced in HU mouse spleens by 59% and, while the T cells number did not change, the Th/Tc ratio was decreased. Finally, the use of a fluorescent dye monitoring lymphoproliferation demonstrated that lymphocyte response to mitogen was reduced in Th and Tc populations and to a greater extent in B cells. Thus, we showed for the first time that, even if restraint has its own effects on the animals and their splenic lymphocytes, the prolonged antiorthostatic position leads, despite the absence of stress, to an inversion of the B/T ratio in the spleen. Furthermore, the lymphoproliferative response was impaired with a strong impact on B cells. Altogether, these results suggest that B cells are more affected by hindlimb unloading than T cells which may explain the high susceptibility to pathogens, such as gram-negative bacteria, described in animal models and astronauts.

## Introduction

Human presence is currently expanding into low Earth orbit and beyond, with the ability to visit space for longer periods of time in the International Space Station and planned missions to Mars. During spaceflight, crewmembers are exposed to various environmental changes, such as confinement, anxiety, microgravity, biomechanical stresses, radiation, disruption of the circadian rhythm and new microbial environments. There have been a large number of studies to understand how this extreme environment affects various physiological functions, especially the immune system [1], [2]. Researchers have demonstrated alterations in innate and specific immunity caused by spaceflight conditions [3], [4], including thymic involution [5], inhibited lymphocyte blastogenesis [6], [7], modification of leukocyte distribution [8], [9] and cytokine production [10]. Moreover, Crucian et al. [11] have recently indicated that this immune dysregulation may depend on mission characteristics such as duration. This was especially true for antibody production, which was not changed during short-term spaceflight [12], while long-term missions showed more severe effects [13], [14]. Furthermore, our previous studies have demonstrated that long-term missions affect immunoglobulin gene expression [15]–[17] and antibody somatic hypermutation frequency [18], [19]. Consequently, before undertaking prolonged space missions, it is important to determine which mechanisms are involved in the spaceflight-induced changes in humoral immunity, which up to now have rarely been investigated.

Due to the limited opportunities to perform space experiments and the reduced number of animals that can be involved in such experiments, it is important to have a ground-based model that reliably mimics the effects of spaceflight. This is why hindlimb unloading was developed. Hindlimb unloading of rodents is a well-accepted ground-based model used to simulate some of the conditions of spaceflight and reproduce its deleterious effects on the musculoskeletal, cardiovascular and immune systems [20]–[22]. This model involves suspending rodents by the tail with a head-down tilt of approximately 25°, which contributes to muscle and bone loss in the hind limbs associated with a fluid shift to the head similar to the changes observed during space missions [23]–[26]. The fluid shift observed in both cases affects the cellular composition of the lymphoid organs and the lymphocyte mitogenic response [27], resulting in a decreased resistance to infections [28]. Studies using this model have also shown complex contradictory observations likely due to differences in animal species, duration, choice of control group, environmental parameters and unloading technique [29]. Most of the experiments were of short duration, between 2 and 10 days [30]–[32], using rats as model. Therefore, it is essential to optimize the suspension conditions in order to compare with spaceflight data. First, as Morey-Holton and Globus [29] have advised, to ensure that the observed changes are not due to tail restraint but to the antiorthostatic position, a restrained control group was added to the control and hindlimb unloaded groups in our experiments. In the restrained group, mice were tail-attached, but the four limbs were allowed to have full contact with the floor of the suspension cage. Secondly, to minimize the contribution of stress to changes in specific immunity in both groups restrained by the tail, we have optimized the Wronsky-Morey-Holton system [25]. This system consists of a wire hooked on a swivel-pulley which allows 360° movement and grooming. Moreover, thymus involution, corticosterone level and body weight were used as indicators of stress. Indeed, studies by Choukèr and others [33] have thoroughly connected physiological stress to changes in specific immune parameters associated with altered gravity. It is also well known that stress hormones significantly influence lymphocyte responsiveness, but whether suspension causes a systemic stress response in mice is less well resolved than for rats. We have recently shown that B cell proliferation is greatly impaired in mice exposed to 21 days of centrifugation without a stress response [34]. In several experiments, the dramatic reduction of splenocytes associated with their depressed proliferative response has been reported [30], [35], whereas the change of the B/T cell ratio and the role of stress has not been discerned. Furthermore, these studies have used traditional *in vitro* methods that take a snapshot of the number of dividing cells by pulsing with tritiated thymidine or measuring the total cell number indirectly [30], [36], [37]. These methods provide information about the broad effects on proliferation but give no deep insight into how the proliferative response is altered. Lyons et al. [38] developed a powerful technique using an intracellular fluorescent label, carboxyfluorescein diacetate succinimidyl ester (CFSE), to analyze the division history of different immunophenotypically defined subpopulations of lymphocytes. The use of CFSE to measure up to eight successive cell divisions by flow cytometry, distinguishing proliferating and non-proliferating populations, has had a great impact on immunological studies.

To better characterize the impact of microgravity on specific immunity, we investigated the effects of long-term exposure to hindlimb unloading in the widely used inbred mouse strain C57Bl/6. The first aim of this study was to determine if restraint was an experimental parameter that had its own influence on immunological parameters. For this purpose, three experimental groups of mice were used: control, restrained and hindlimb unloaded. The second aim was to determine whether the effects of antiorthostatic suspension on the immune system can be studied apart from the effects of the stress response. Thus, thymus and spleen weights and serum corticosterone concentrations were measured. The third aim was to assess the different lymphocyte sub-populations in the spleen and their mitogenic response *in vitro*. For that purpose, we used cell staining markers, fluorescent antibodies to identify lymphocyte phenotypes and CFSE to quantify divided cells. Finally, cytokine secretion was quantified after *in vitro* stimulation.

## Materials and Methods

### Ethics statement

All manipulations with animals were carried out in accordance to the National Legislation and the Council Directive of the European Communities on the Protection of Animals Used for Experimental and Other Scientific Purposes (2010/63/UE). This protocol has been approved by the regional ethic committee: Comité régional d'éthique pour l'expérimentation animale – Comité régional d'éthique pour l'expérimentation animale: Nancy - Lorraine - Nord-Est (number agreement: CELMEA-2012-0008).

### Animals

Experiments were conducted on eight-week-old C57Bl/6N-Crl male mice of a mean body weight of 20 g purchased from Charles River Laboratories (France). Animals were housed in vented animal cabinet (Noroit, Bouaye, France) under controlled temperature and a 12 h∶12 h dark/light cycle with food and water *ad libitum*. They were allowed to rest in groups of four mice in standard cages (35 cm deep × 15 cm wide × 14 cm high) for one week following arrival before being isolated in a random way for the experiments.

### Hindlimb unloading

Mice were isolated five days before the beginning of the experiments in standard or specific cages. Each group consisted of 4 to 6 mice for each experiment. The control group (C) was housed in standard cages (35 cm deep × 15 cm wide × 14 cm high) while hindlimb unloaded (HU) and restrained (R) groups were housed in cages manufactured according to Chapes et al. [27] (35 cm deep × 15 cm wide × 26 cm high). Mice were suspended by using a dressing retention sheet wrapped around the tail and a wire hooked on a swivel-pulley system. To prevent mice from chewing, a small plastic tube was fixed onto the traction tape with small strips of medical paper tape. The swivel pulley glides along two stainless steel rods that run the length of the cage, which provides a full 360° range of movement. The angle of suspension for HU mice was adjusted to 25–30° such that only the forelimbs touch the grid at the bottom of the cage. Orthostatic restrained mice were not suspended, so all limbs were in contact with the grid. Throughout the 21 days of experimentation, mice were weighed daily during the first four days, then every two days thereafter. All experiments were repeated at least three times.

### Sample collection

On day 21, mice were weighed and then euthanized by decapitation. Trunk blood was collected and allowed to clot at ambient temperature for 15 min. Serum was collected after a centrifugation at 2000 g at 4°C for 15 min and stored at −80°C before corticosterone and cytokine quantification. The thymus and spleen of each animal were removed and weighed. The spleen was placed immediately into sterile tubes containing 3 ml of RPMI 1640 medium (PAA, Pashing, Austria) to perform lymphocyte assays.

### Lymphocyte populations

The spleen was dissociated into RPMI 1640 medium. Red blood cells were lysed with NH_4_Cl 140 mM (eBioscience, San Diego, CA, USA) before counting nucleated cells using a hemocytometer. To evaluate lymphocyte sub-populations, 10^6^ splenic cells were incubated for 15 min at 4°C in the dark with a mixture of four anti-mouse fluorescence-labeled monoclonal antibodies: ECD anti-CD19 (clone 6D5, Beckman Coulter, Marseille, France), APC anti-CD3ε (clone 17A2, eBioscience), PE anti-CD4 (clone RM4-5, eBioscience) and PE-Cy7 anti-CD8α (clone 53-6.7, eBioscience). Immunophenotyping was carried out using a five-color FC500 flow cytometer (Beckman Coulter). A first gate was applied to select the lymphocyte population using a window with forward scatter (FSC) and side scatter (SSC). Fifty thousand events in the viable lymphocyte gate were acquired. Data were analyzed using FlowJo software v7.6.5 (Tree Star Inc., Ashland, OR, USA) to identify specific lymphocyte sub-populations: CD19^+^ B cells, CD3^+^ T cells, CD3^+^CD8^+^ cytotoxic T cells (Tc) and CD3^+^CD4^+^ helper cells T (Th). Populations are expressed as percentages of the total lymphocyte population.

### Lymphoproliferation measurement

To evaluate lymphocyte proliferative responses, 20×10^6^ splenic cells were suspended in 1 ml of Phosphate Buffer Saline (PAA) supplemented with 1% heated fetal calf serum (FCS, Sigma-Aldrich, Saint-Louis, MO, USA). CFSE (Life Technologies, Paisley, UK) was added to a final concentration of 5 μM in DMSO (dimethyl sulfoxide) and the suspension was incubated at 37°C for 8 min. The staining was quenched with 2 ml of ice-cold FCS and incubated for 5 min at 4°C. Cells were collected by centrifugation and washed in culture medium, RPMI 1640 medium supplemented with 10% heated FCS, 100 U penicillin, 100 μg/ml streptomycin and 2 mM glutamine (Sigma-Aldrich). Finally, cells were adjusted to 10×10^6^/ml in culture medium. Two mitogens, lipopolysaccharide (LPS) from *Escherichia coli* (Sigma-Aldrich) and concanavalin A (ConA, Sigma-Aldrich), were used to stimulate lymphocytes. Cells were dispensed in 50 μl triplicates into the wells of a 96-well round-bottom tissue culture plate containing 50 μl of culture medium without (unstimulated cells) or with mitogen (stimulated cells) at a final concentration of 5 μg/ml. The plate was incubated for 72 h at 37°C and 5% CO_2_. After 72 h, cells from each well were washed in PBS before being incubated with the mixture of fluorescent antibodies used for phenotyping. The supernatants from each well were frozen at −80°C until cytokine quantification. All fluorochrome-stained lymphocyte cultures were analyzed by five-color analysis using the FC500 flow cytometer. The lymphocyte gate was based on light scatter parameters, cell size and internal granularity. Thus, the gate was placed on the population that represented the viable lymphocytes plus the blast cells, which are generally low on side scatter. Within this gate, 50,000 events were acquired and the proportions of each cell type (CD19^+^ B cells, CD3^+^ T cells, CD3^+^CD4^+^ helper T cells (Th), CD3^+^CD8^+^ cytotoxic T cells (Tc)) were determined using FlowJo software v7.6.5. To calculate the percentages of increase of each lymphocyte sub-population in response to mitogen stimulation, the following formula was used: [(% population stimulated - % population unstimulated)/% population unstimulated]. The fluorescence of CFSE staining was measured to determine the proportion of divided cells among total lymphocytes or cell subsets focusing on CD19^+^ cells after stimulation with LPS or on CD3^+^CD8^+^ and CD4^+^ cells after stimulation with ConA. The FlowJo software determines generation 0, meaning the cells with 100% of max CFSE, and calculates the percentage of divided cells from this, using the number of events and CFSE fluorescence.

### Corticosterone assay

The corticosterone concentration was measured without any extraction procedure using a commercial ELISA kit according to the manufacturer's instructions (Arbor Assays, Ann Arbor, MI, USA). The concentration of corticosterone in serum samples was calculated using a 4-parameter curve and expressed in ng/ml. The intra- and inter-assay coefficients of variation were under 8.4% and 13.1%, respectively.

### Cytokines analysis

Mouse sera and culture supernatants were thawed immediately before analysis. Cytokines were quantified using the Mouse Th1/Th2 10plex FlowCytomix Kit (eBioscience) according to the manufacturer's instructions using the FC500 flow cytometer. The concentration of each cytokine was calculated using the FlowCytomix Software (eBioscience) and expressed in pg/ml. The limits of detection of each analyte were: GM-CSF 10.9 pg/ml, IFN-γ 6.5 pg/ml, IL-1α 15.7 pg/ml, IL-2 8.8 pg/ml, IL-4 0.7 pg/ml, IL-6 2.2 pg/ml, IL-5 4.0 pg/ml, IL-10 5.4 pg/ml, IL-17 2.4 pg/ml and TNF-α 2.1 pg/ml.

### Statistics

SPSS v13.0 (SPSS Inc, Chicago, IL, USA) was used to perform statistical analyses. Outlier values were determined by a boxplot of each group studied. Once normality and homogeneity of variances were assessed as determined by Kolmogorov-Smirnov and Levene tests, an ANOVA was performed and a Tukey post-hoc test was used to establish inter-group comparisons (C, R and HU). When the data were not normally distributed, a Kruskal-Wallis non parametric test was performed and a Dunnett T3 post-hoc test was used for inter-group comparisons. To indicate significance and trend, p-values of <0.05 and <0.10 were selected, respectively. All data are presented as the means with standard error of the mean (SEM).

## Results

### Body weight

**Figure 1:**
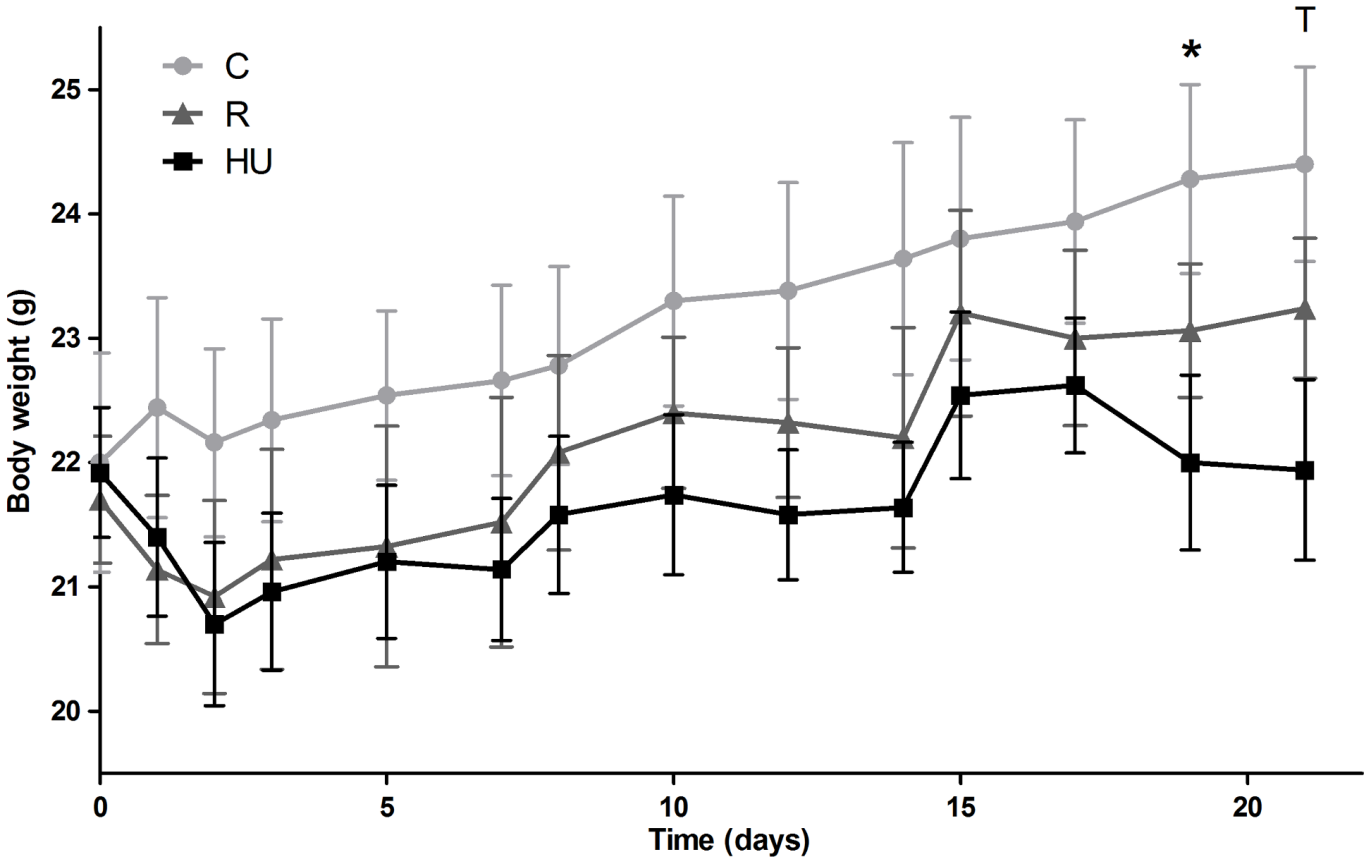
Body weight evolution in control (C), restrained (R) and hindlimb unloaded (HU) mice. Mice of each group were weighed daily for the first four days and then every two days. Every seven days, cages were cleaned and feeders and bottles were refilled with food and water. Weights are presented with SEM for each condition of housing. Each experimental group was compared to the others. *n* = 5 mice per group. Differences were found to be statistically significant using ANOVA and a Tukey *post-hoc* test. * *p* = 0.035 HU *versus* C by day 19; ^T^ *p* = 0.068 HU *versus* C by day 21.

Because differences in body weight between the HU and C groups can influence the interpretation of the experimental results, we recorded the weights of the mice during the 21 days of experimentation. The C mice grew regularly while both R and HU mice exhibited a significant drop in body weight during the first three days, but no further decrease was observed over the next days (Fig. 1). R mice recovered their initial weight on day 8, whereas HU mice returned to their initial weight on day 15. The mean body weight for the HU group was significantly lower on day 19 (*p* = 0.035) than that observed in the C group, and a tendency toward decreased weight (*p* = 0.068) was observed on day 21. Inter-individual variability, revealed by standard error bars, was large but similar for all groups. This is most likely due to the young age of the mice, because their weight was not yet constant. Two peaks of increase were observed at days 8 and 15 for the R and HU groups. These peaks were concomitant with litter cleaning at days 7 and 14 coupled with the refilling of food and water. At the end of the experiment, body weight gain was not significantly different (*p* = 0.263) for the R and C mice, which gained an average of 1.5 g and 2.4 g, respectively (Table 1). A significant difference appeared when HU mice were compared to R and C mice (*versus* R: *p* = 0.032; *versus* C: *p* = 0.002), as they did not gain weight, while the other groups grew by approximately 7% (R) and 11% (C) in comparison to their initial weights.

**Table 1:** Body weight gain, lymphoid organ normalized weight and spleen cellularity in control (C), restrained (R) and hindlimb unloaded (HU) mice at the end of the experiments.

Group	Body weight gain (g)		Normalized spleen weight (mg/g)		Normalized thymus weight (mg/g)		Number of splenic nucleated cells (x10^6^)	
C	**+2.40**	±0.290	**3.04**	±0.047	**3.10**	±0.327	**73.9**	±5.8
R	**+1.54**	±0.334	**2.91**	±0.212	**2.66**	±0.245	**60.1**	±10.6
HU	**+0.02**	±0.460* ^,¤^	**3.06**	±0.208	**2.74**	±0.220	**55.1**	±7.4

C  =  Control, R  =  Restrained, HU  =  Hindlimb Unloaded.

* *p* = 0.032 *versus* R, ^¤^ *p* = 0.002 *versus* C.

Body weight gain was calculated with the formula [body weight on day 21 - body weight on day 0 of the treatment]. Normalized weights were calculated with the ratio [organ weight/body weight] for each mouse. The number of splenic nucleated cells was calculated after red blood cell lysis with NH_4_Cl. Each group was compared to the others. *n* = 5 mice per group. Differences were found to be statistically significant using an ANOVA and Tukey *post-hoc* test. HU mice did not gain weight (*p* = 0.032 *versus* R, *p* = 0.002 *versus* C), while the other groups grew by approximately 7% (R) to 11% (C) in comparison to their initial weight. No significant difference was found between the three experimental groups for lymphoid organ normalized weights. The number of nucleated cells was reduced by 19% and 25%, respectively, in the R and HU mice in comparison to C mice, although these differences were not significant (*versus* R: *p* = 0.479; *versus* HU: *p* = 0.273). Data are mean values ± SEM.

### Lymphoid organs

To assess the stress and immunological status of the mice at the end of the experiments, we measured the thymus and spleen weights and normalized these values to body weights (Table 1). No significant difference appeared between the normalized spleen weights of the three groups of mice. The number of nucleated cells in the spleen was reduced in the R and HU mice by 19% and 25%, respectively, although these differences were not statistically significant (*versus* R: *p* = 0.479; *versus* HU: *p* = 0.273) (Table 1). Although a slight decrease was observed in the normalized thymus weight in the R and HU mice, it was not significant when compared with the C group (*F* = 0.742, *p* = 0.497). Notably, the same trend was observed in all previous suspension experiments we have performed.

### Serum corticosterone concentration

**Figure 2:**
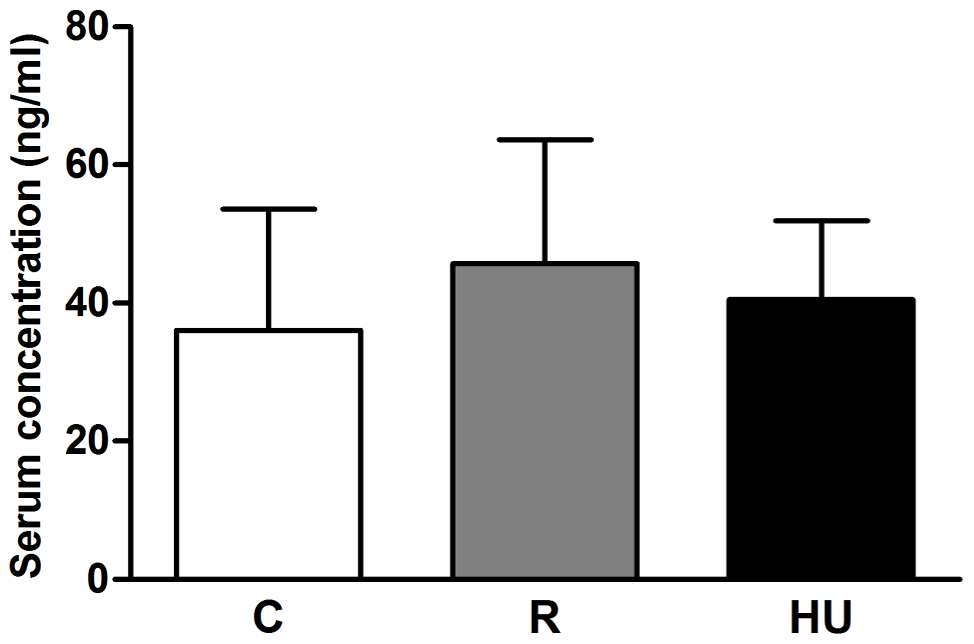
Serum corticosterone concentration in control (C), restrained (R) and hindlimb unloaded (HU) mice. Trunk blood was collected immediately after the sacrifice. Corticosterone concentration was measured using an ELISA kit with a detection threshold of 16.9/ml. Each experimental group was compared to the others. *n* = 4, 6 and 4 mice for C, R and HU groups, respectively. No significant difference was found using an ANOVA and Tukey *post-hoc* test.

To evaluate the stress response, we measured the concentration of corticosterone in the serum of each mouse in addition to measuring the weight of the thymus. After 21 days of experimentation, there was no difference between the corticosterone concentrations of the three groups (Fig. 2). All of the concentrations were below 50 ng/ml, suggesting that the mice were adapted to the orthostatic or antiorthostatic conditions. Again, it is noteworthy that the same trend was observed in all previous suspension experiments we performed.

### Splenic lymphocytes populations at the end of the treatments

**Figure 3:**
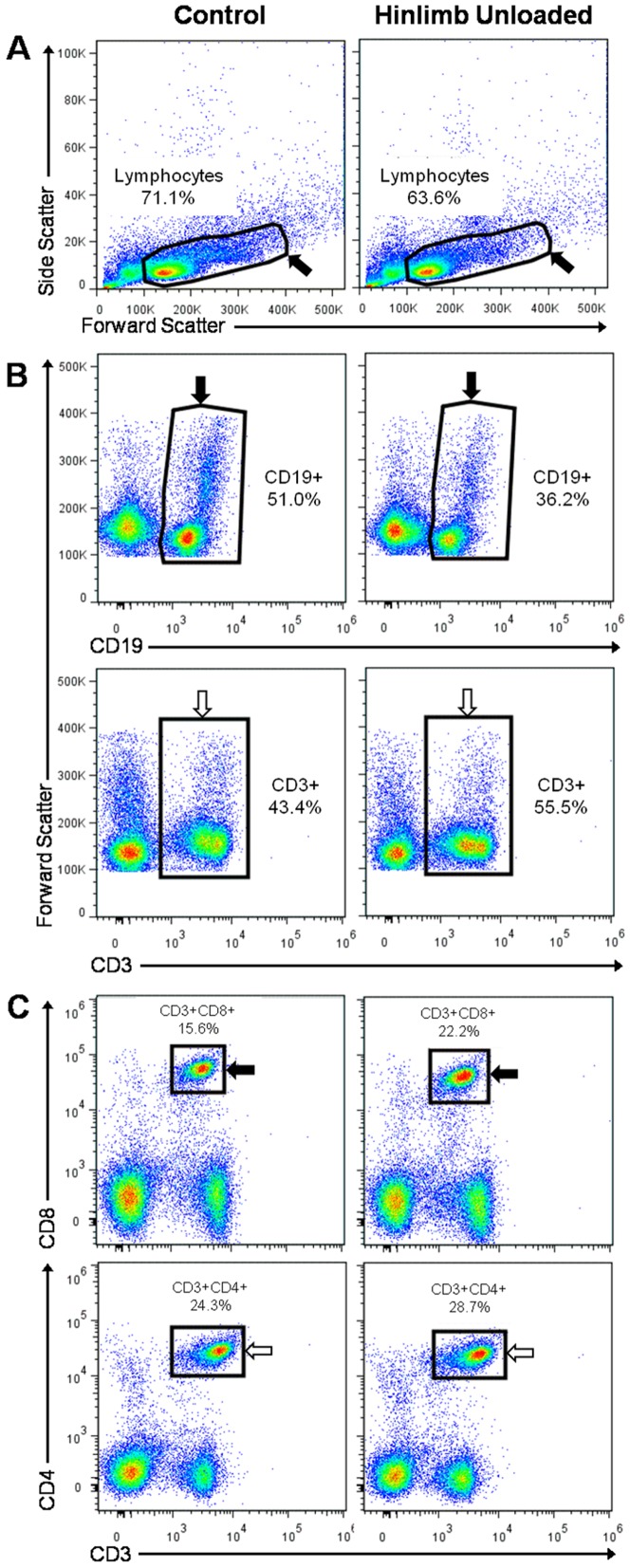
Representative flow cytometry density plots from splenic lymphocyte populations. Splenocytes were isolated from mice and labeled as described in the Methods section. For each mouse, the lymphocyte populations were determined as in the two examples presented here. (**A**) Splenocyte analysis by flow cytometry using FlowJo software and sorted with FSC/SSC profiles, separating viable lymphocytes (black arrow) from the other cells. (**B**) Among the lymphocyte population, CD19^+^ B cells (black arrow) were selected in the FSC/CD19 window and CD3^+^ T cells (white arrow) were selected in the FSC/CD3 window. (**C**) Among the lymphocyte population, CD3^+^CD8^+^ T cells (black arrow) were selected in CD3/CD8 profiles and CD3^+^CD4^+^ T cells (white arrow) were selected in CD3/CD4 profiles.

**Figure 4:**
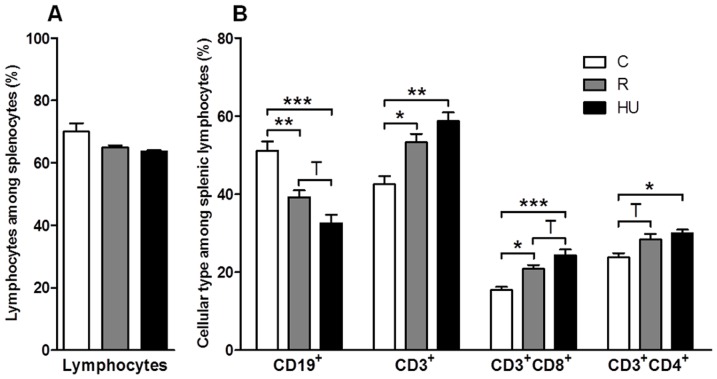
Phenotypes of splenic lymphocytes in control (C), restrained (R) and hindlimb unloaded (HU) mice. After incubation with fluorescent antibodies, cell populations were identified by flow cytometry. (**A**) Viable lymphocytes were first gated using a forward and side scatter window. (**B**) Populations of interest were then expressed as percentages of the lymphocyte population. Each experimental group was compared to the others. *n* = 4, 6 and 4 mice for C, R and HU groups, respectively. Differences were found to be statistically significant using an ANOVA and Tukey *post-hoc* test. ^T^ *p*<0.1; * *p*<0.05; ** *p*<0.005; *** *p*<0.001. The comparison of HU and C groups reveals that CD19^+^ B cells are decreased by 40% (p<0.001) while CD3^+^ T cells are increased by 38% (p = 0.002) in HU mice. Both CD3^+^CD8^+^ and CD3^+^CD4^+^ subsets are significantly more abundant in HU than in C mice (by 60% and 25%, respectively).

To determine how orthostatic and antiorthostatic restraints modify the number and the proportions of B and T cell subpopulations in the spleen, we carried out an immunophenotyping of the lymphocytes. The different sub-populations were determined within the viable lymphocyte gate (Fig. 3A). No statistically significant difference was observed between the total lymphocyte percentages for the three groups of mice which was 70.0% for C group and 64.9% and 63.8% for R and HU groups respectively (Fig. 4A), although the number of nucleated cells was decreased by 25% for the HU group (Table 1). To determine to what degree loss or increase of total lymphocytes and each subset contribute to the percentage changes reported, the absolute numbers were calculated from the number of nucleated cells (Table 2). The number of total lymphocytes decreased in R and HU groups (25% and 33% respectively), although these decrease were not statistically significant.

**Table 2:** Absolute numbers of lymphocytes and lymphocytes subsets in the spleen (x10^6^).

Group	Lymphocytes		CD19^+^		CD3^+^		CD3^+^CD8^+^		CD3^+^CD4^+^	
C	**52.3**	±6.3	**27.1**	±4.2	**22.0**	±2.1	**8.0**	±0.7	**12.3**	±1.2
R	**39.0**	±6.9	**15.2** *	±2.7	**21.1**	±4.1	**8.2**	±1.5	**11.4**	±2.4
HU	**35.1**	±6.2	**11.1^¤^**	±1.3	**21.0**	±4.4	**8.8**	±2.0	**10.7**	±2.1

C  =  Control, R  =  Restrained, HU  =  Hindlimb Unloaded.

* *p* = 0.039 *versus* C; ^¤^ *p* = 0.011 *versus* C.

Absolute numbers for each cell population was calculated using the number of splenic nucleated cells and the percentages obtained by flow cytometry. Each group was compared to the others. *n* = 4, 5 and 4 mice for C, R and HU groups, respectively. Differences were found to be statistically significant using an ANOVA and Tukey *post-hoc* test. The number of total lymphocytes was reduced by 25% and 33%, respectively, in R and HU mice in comparison to C mice. There was no significant difference for T cells and their subpopulations for the three groups. The number of B cells was significantly decreased by 44% (*p* = 0.039) and 59% (*p* = 0.011) in R and HU mice, respectively. Data are mean values ± SEM.

To quantify the proportions of lymphocyte subpopulations, positive cells for each phenotypic marker were selected in the lymphocyte gate. CD19^+^ was used for B cells, CD3^+^ for T cells (Fig. 3B), CD3^+^/CD8^+^ for cytotoxic T cells and CD3^+^/CD4^+^ for helper T cells (Fig. 3C). This cytometric analysis was performed on mice from each of the 3 groups, and results are shown in Figure 4B. CD19^+^ B cells represent 51.2% of total lymphocytes in the spleen of C mice, compared to 39.2% and 32.6% in the spleen of R and HU mice, respectively. Thus, there is a decrease in B cells of 23% in the R group and 36% in the HU group. Both differences were statistically significant, with a greater difference in the antiorthostatic restrained mice (C *versus* R: *p* = 0.003; C *versus* HU: *p*<0.001). Moreover, the absolute number of B cells was significantly decreased by 44% and 59% in R and HU groups respectively (*p* = 0.039 and *p* = 0.011) when the proportions were related to total lymphocytes and compared to C group (Table 2). Figure 4B shows that in the R and HU groups, the percentages of total T cells (CD3^+^) were significantly increased by 25% and 38%, respectively (C *versus* R: *p* = 0.011; C *versus* HU: *p* = 0.001). Even if the proportion of T cells was raised compared to B cells in R and HU groups, the absolute numbers of T cells did not change (Table 2). However, the B/T ratio dramatically decreased from 1.2 to 0.7 and 0.5 after 21 days of orthostatic and antiorthostatic restraints respectively, both in percentages and absolute numbers. In the spleens of the HU mice, the percentages of CD3^+^CD8^+^ and CD3^+^CD4^+^ T cells were increased by 57% (*p*<0.001) and 26% (*p* = 0.021), respectively (Fig. 4B) but the absolute numbers of these subpopulations did not significantly vary between the 3 groups (Table 2). However, the CD4^+^/CD8^+^ ratio was reduced for R and HU mice both in percentages and absolute numbers. This ratio was 1.5 for C mice, compared to 1.4 and 1.2 for the R and HU groups, respectively. A close inspection of the perturbations measured in the lymphocyte populations showed that hindlimb unloading significantly worsened the modifications in the lymphocyte populations observed during orthostatic restraint.

### Variations of splenocytes populations 72 hours after *in vitro* stimulation

**Figure 5:**
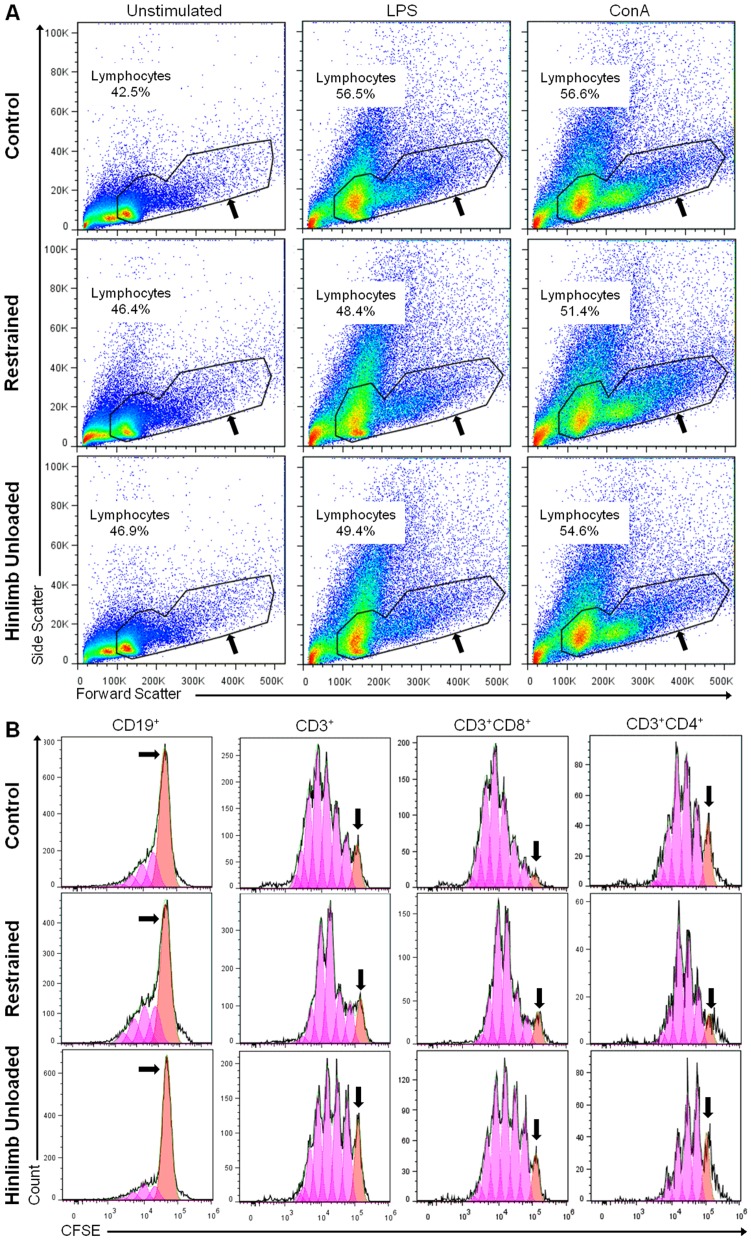
Detection of lymphocyte division after 72*in vitro* stimulation with or without mitogen. Splenocytes were labeled with CFSE and cultured for 72(LPS or ConA). For each mouse, dividing lymphocytes were determined as in the three examples presented here. (**A**) Cells were labeled with fluorescent antibodies and analyzed by flow cytometry. They were sorted with FSC/SSC profiles, separating viable lymphocytes (black arrow) from dead cells and cellular debris. (**B**) Among the viable lymphocyte gate, each subpopulation was selected as described in Figure 3 and assessed for fluorescent division peaks. Generation 0 (black arrow) and each of the following generations (pink area) were used by the software to calculate the number of dividing cells.

**Figure 6:**
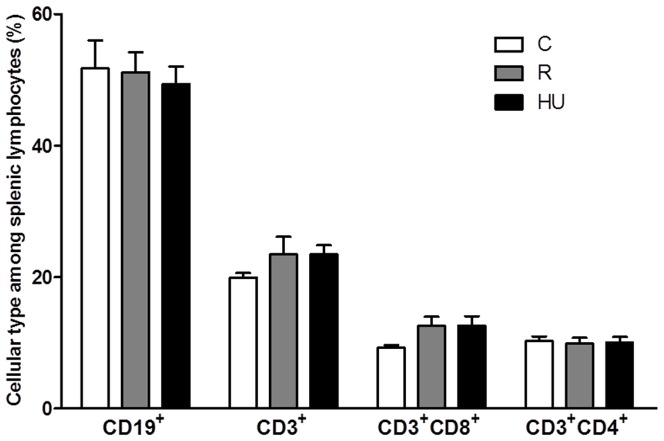
Phenotypes of splenic lymphocytes from control (C), restrained (R) and hindlimb unloaded (HU) mice after 72 h of incubation without mitogen. After incubation with fluorescent antibodies, cell populations were identified by flow cytometry. Lymphocytes were first gated according to their size and granularity. Populations of interest were then expressed as percentages of the lymphocyte population. Each experimental group was compared to the others. *n* = 5 mice per group. No significant difference was found using an ANOVA and Tukey *post-hoc* test.

After 72 hours of *in vitro* stimulation with different mitogens, cells were analyzed by flow cytometry. Figure 5A presents an example of the analysis. First, a viable lymphocyte gate was applied to select all cells, not only those that responded to the stimulation. Indeed, granularity levels increase when cells die, so they could be excluded using SSC. Each subset was selected from the viable lymphocytes as described in Figure 3. Because the proportions of lymphocyte sub-populations were different between the three experimental groups at the end of the treatment (Fig. 4B) and after 72 h of culture without mitogen (Fig. 6), to precisely compare the mitogenic-induced blastogenesis, we calculated the percentage of increase or decrease for each cell type after 72 h of incubation using the formula described in the Materials and Methods section. These calculations were performed for each mouse of the three groups and the results are presented in Table 3.

**Table 3:** Variations of lymphocyte populations 72

	LPS Stimulation		ConA stimulation			
Group	Lymphocytes	CD19^+^	Lymphocytes	CD3^+^	CD3^+^CD8^+^	CD3^+^CD4^+^
C	**+41.6%**±3.3	**+12.6%**±8.1	**+40.0%**±2.1	**+73.7%**±19.3	**+139.4%**±22.1	**−2.2%**±15.7
R	**+31.9%**±7.0	**+9.4%**±2.2	**+36.3%**±6.4	**+73.0%**±11.8	**+87.1%**±26.2	**−22.9%**±8.6
HU	**+16.9%** *±6.2	**+10.2%**±4.7	**+29.3%**±3.5	**+43.8%**±12.3	**+61.3%^¤^**±20.7	**−22.0%**±14.0

C  =  Control, R  =  Restrained, HU  =  Hindlimb Unloaded.

* *p*  =  0.025 *versus* C. ^¤^ *p*  =  0.081 *versus* C.

After 72 hours of culture, cells were incubated with fluorescent antibodies before identification by flow cytometry. Specific subpopulations of lymphocytes were determined within the lymphocyte gate. The formula [(% population stimulated - % population unstimulated)/% population unstimulated] was used to calculate the percentage of variation after stimulation with mitogens. Each group was compared to the others. *n*  =  5 mice per group. Statistically significant differences were found using an ANOVA and Tukey *post-hoc* tests. After LPS stimulation, the number of total lymphocytes was increased by only 16.9% in the hindlimb unloaded (HU) group compared to 41.6% in control (C) mice, corresponding to a reduction of 60% that was significantly different (*p*  =  0.025 *versus* C). Data are mean values ± SEM.

The increase in total lymphocytes after 72 h of stimulation with LPS for both R and HU mice was lower compared to the C mice (Table 3). The increase of total lymphocyte percentage was reduced in the mice from the HU group (16.9%), compared to their control counterpart from the C group (41.6%). These differences correspond to a significant 60% reduction of the increase of the total lymphocyte percentages in HU mice compared to the C group (*p* = 0.025). For the R group, the mitogenic-induced blastogenesis was also lower than the C group but statistically non-significant. When we focused on the CD19^+^ population, the slight decreases observed for the HU and R groups compared to the C group were not significant.

After ConA stimulation, no significant difference was observed between the three experimental groups (Table 3), even if the percentage of increase in total lymphocytes was higher for the C group (+40.0%) than for both R and HU groups (+36.3% and +29.3%, respectively). There was no difference between the C and R groups in the response of CD3^+^ cells to ConA (+73.7% and +73.0%, respectively), whereas there was a 40% decrease of CD3^+^ cells response when the HU group was compared to C mice. In the CD3^+^ cell subsets (CD4 and CD8), there were differences between the C group and both restraint groups, but these were not statistically significant. The percentage of increase in CD3^+^CD8^+^ cells after stimulation was reduced by 56% for the HU mice (tendency, *p* = 0.081) and 37% for the R mice compared to C mice (Table 3). Surprisingly, the CD3^+^CD4^+^ cell population decreased by 22% in both the R and HU groups after 72 h of ConA stimulation, while for C mice almost no increase was detected (Table 3). This could be explained by an unresponsiveness of these cells to mitogen, or by their proliferation being immediately followed by death, as we can see in Figure 5A. Thus, it appears that the two T cell subpopulations, Th and Tc, were not affected in the same way by the suspension. Moreover, if we consider only the proliferative response of the total lymphocytes, we can see that the effect of the antiorthostatic position increased the negative effect of attachment. However, the results are more subtle when we look at the level of lymphocyte subsets.

### Splenocytes proliferation after 72 hours of *in vitro* stimulation

To determine the extent to which the different lymphocyte subsets were affected, CFSE labeling was used to track cell proliferation profiles. In the example presented in Figure 5B, the number of generations calculated by the FlowJo software was similar between experimental groups, except for Th cells. For CD19^+^ B cells, there were only 4 generations, and many cells remained in generation 0. For CD3^+^ T cells, 7 generations were observed after 72 h of stimulation with ConA, except for the CD3^+^/CD4^+^ Th cells of HU mice. Moreover, the number of T cells in generation 0 was lower than the number of B cells in generation 0. For all of the subsets, the repartition of daughter cells between generations was different between HU and C mice.

**Figure 7:**
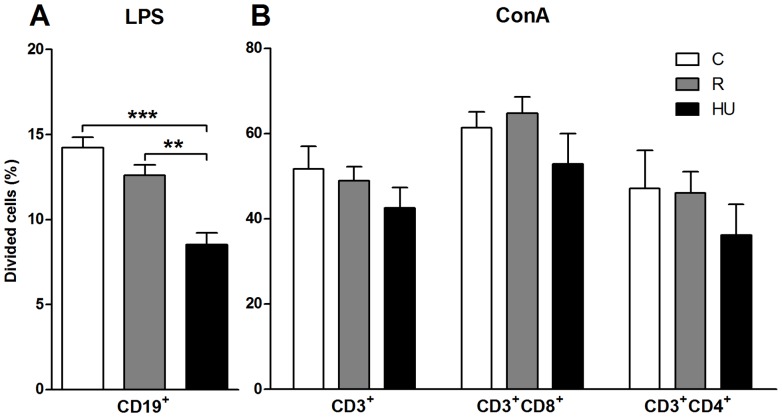
Percentages of dividing lymphocytes 72 hours after mitogenic stimulation. *In vitro* culture of splenocytes stained with CFSE allowed the analysis of B, Tc and Th divisions in response to a mitogenic stimulation. Subpopulations of lymphocytes were determined within the lymphocyte gate. Within each subpopulation, CFSE fluorescence was analyzed by flow cytometry. Then, the FlowJo software automatically determined generation 0 and calculated the percentage of dividing cells from the intensity of residual CFSE in cells. Each group was compared to the others. *n* = 5 mice per group. Differences were found to be statistically significant using an ANOVA and Tukey *post-hoc* test. ** *p*<0.005; *** *p*<0.001. (**A**) Percentages of dividing CD19^+^ B cells in response to LPS. Dividing B cells were significantly decreased in the hindlimb unloaded (HU) group compared to the restrained (R) and control (C) groups (by 33% and 40%, respectively). (**B**) Percentages of dividing CD3^+^ T cells, CD3^+^CD8^+^ Tc cells and CD3^+^CD4^+^ Th cells in response to ConA. No significant difference was obtained for dividing T cells despite the decreases observed for the HU group compared to C mice.

The total number of dividing cells in each subset was calculated for each mouse. Mean values are presented in Figure 7. Data from the 3 groups of mice confirm that the percentage of dividing CD19^+^ cells in response to LPS was lower than the percentage of dividing CD3^+^ cells in response to ConA (10–15% with LPS versus 36–65% for ConA). As observed in table 3, orthostatic restraint had a slight, but not significant, effect on the proliferation of lymphocytes stimulated either with LPS or ConA. Compared to the C group, restraint induced a 5% decrease in the number of T cells (CD3^+^) that divided in response to ConA and a 12% decrease in B cells (CD19^+^) that divided in response to LPS (CD3^+^: *p* = 0.907; CD19^+^: *p* = 0.213). Note that the numbers of dividing helper and cytotoxic T cells were similar between the R and C groups (*p* = 0.993 and 0.886, respectively).

Even if the percentage of dividing cells was low after LPS stimulation, our experiments show that HU has a negative effect on the mitogenic response of splenocyte subpopulations. Indeed, the number of dividing CD19^+^ cells after LPS stimulation was decreased by 32% (*p* = 0.002) and 40% (*p*<0.001) in HU mice when compared to the R and C groups, respectively (Fig. 7A). The number of dividing CD3^+^ cells from HU mice was decreased by 13 and 18% when compared to the R and C groups, respectively (Fig. 7B). Moreover, each CD3^+^ subpopulation, CD3^+^CD4^+^ and CD3^+^CD8^+^, was not affected in the same proportion. The response of cytotoxic T cells from the HU group was decreased by 14% in comparison to the C group, whereas there was a 23% reduction in helper T cells. Unfortunately, these data were not statistically significant.

Collectively, these findings indicate that hindlimb unloading by itself has a great negative impact on the *in vitro* responsiveness of splenic lymphocytes, principally targeted to the B cell population.

### Cytokines secretion after 72 hours of *in vitro* stimulation

**Figure 8:**
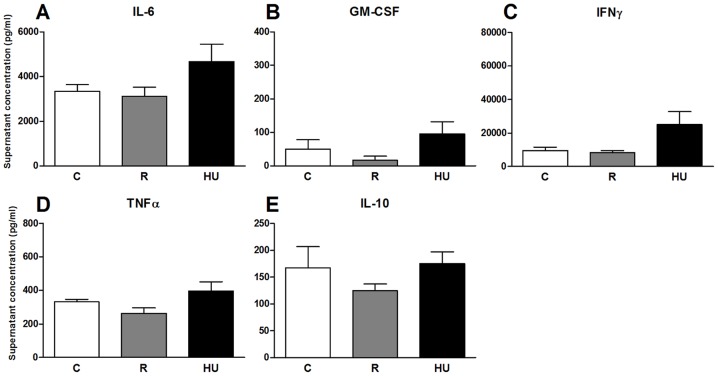
Cytokines secreted by splenic lymphocytes stimulated with LPS. Splenocytes were incubated with 5 μg/ml of LPS for 72 h. Cytokine concentrations in the supernatants were determined by flow cytometry using a Flowcytomix kit. Each group, hindlimb unloaded (HU), restrained (R) and control (C), was compared to the others. *n* = 5 mice per group. No significant difference was found between the three experimental groups using ANOVA and Tukey *post-hoc* test. Cytokines whose concentrations were below the detection threshold of the kit are not indicated.

First, it should be noted that the detection threshold of the kit used to quantify cytokines in the serum of mice was not low enough to detect differences between the 3 groups of mice. Thus, unlike previous reports concerning centrifuged mice or astronauts, hindlimb unloading or restraint did not induce a detectable increase of the 10 measured cytokines in peripheral blood. Consequently, to determine whether the negative effect of restraint or hindlimb unloading on the proliferative response resulted in the differential activation of cytokine responses, secreted cytokines were measured in culture supernatants after 72 h of lymphocyte culture with LPS or ConA. Ten cytokines were measured in the supernatants of cells stimulated with LPS, but concentrations of IL-1α, IL-2, IL-4, IL-5 and IL-17 were below the detection threshold (15.7, 8.8, 0.7, 4.0 and 2.4 pg/ml, respectively) of the kit. Figure 8 shows the concentrations of the other five cytokines. Whereas there were no statistically significant differences between the three groups, there were some changes between the HU and both the C and R groups, depending on the cytokine. IL-6 was slightly increased in HU mice, with no change in R mice (Fig. 8A). GM-CSF was increased two-fold in HU mice (Fig. 8B). The secretion of IFN-γ was strongly increased in HU mice, with no change between C and R mice (Fig. 8C). There were no changes in the TNF-α and IL-10 concentrations between the C and HU mice (Fig. 8D and 8E).

**Figure 9:**
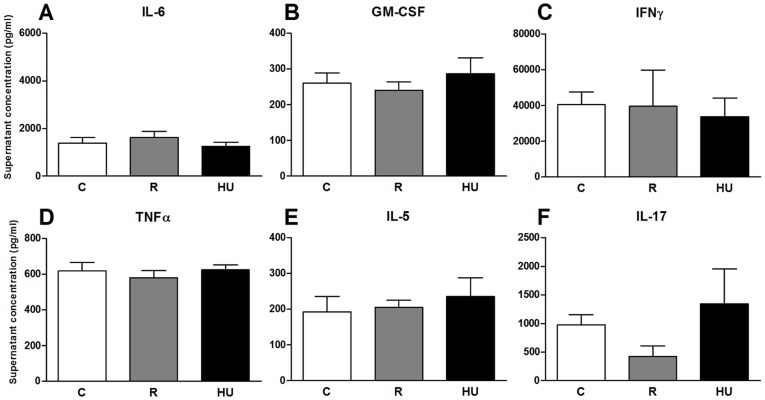
Cytokines secreted by splenic lymphocytes stimulated with ConA. Splenocytes were incubated with 5 μg/ml of ConA for 72 h. Cytokine concentrations in the supernatants were determined by flow cytometry using a Flowcytomix kit. Each group, hindlimb unloaded (HU), restrained (R) and control (C), was compared to the others. *n* = 5 mice per group. No significant difference was found between the three experimental groups using ANOVA and Tukey *post-hoc* test. Cytokines whose concentrations were below the detection threshold of the kit are not indicated.

Figure 9 shows the concentrations of the six cytokines that could be quantified in the culture supernatants after 72 h of stimulation with ConA. IL-1α, IL-2, IL-4 and IL-10 concentrations (sensitivity of 5.4 pg/ml) were under the detection threshold. For the other cytokines, no significant differences were observed between the three experimental groups. Only the IL-17 concentration was two-fold lower in R mice compared to the C and HU mice (Fig. 9F), but the SEM was high and thus must be interpreted cautiously.

## Discussion

Our results show that both R and HU mice lost weight during the first three days of the experiments, but they adapted quickly to the treatments, as indicated by increasing body weight starting on the fourth day (Fig. 1). The R mice gained weight in a similar manner to the C group at the end of the experiments, while the HU mice just recovered their initial weight. Other investigators have shown that HU rats also experienced a significant reduction in body weight, similar to flown rats [39]–[41]. This weight reduction is most likely induced by the decrease of food intake at the beginning of the suspension, as previously described by Morey-Holton and Globus [29]. This hypothesis is sustained by the observation of two small increases in body weight after the provision of new food and water on days 7 and 14 (Fig. 1). Moreover, Tsvirkun et al. [42] have observed an increase in locomotor activity during the second half of rat unloading experiments, and Momken et al. [43] have reported an increase in total energy expenditure in rats unloaded for 14 days. These observations could also account for the lower body weight of HU mice.

In this study, hindlimb unloading did not alter the normalized weights of the spleen and thymus. Our data are in agreement with Felix et al. [21], who observed no difference between the lymphoid organ weights of C and HU mice. However, Caren et al. [44] noticed splenic atrophy after 22 days of rat suspension. On the whole, the effect of gravity modification on lymphoid organ weight has been reported to be variable [40], [45], [46]. This could be explained by the variability of experimental conditions, including the spaceflight profile, ground-based model, duration and animal model used. Furthermore, in their review, Morey-Holton and Globus [29] have thoroughly described the importance of controlling the environmental parameters and using an adapted tail traction system to maintain a low level of stress for the mice. Numerous studies have demonstrated a close correlation between elevated corticosterone concentration and the weight of lymphoid organs after hyper- or microgravity exposure [47], [48]. In our experiments, the corticosterone concentration was the same in the three groups of mice (Fig. 2). It is likely that in the early days of our experiment, the concentration increased, as observed by Steffen and Musacchia [49], because both restraint and tail suspension are stressful events. Nevertheless, in our hands, the mice adapted quickly to the suspension, most likely because of the improved attachment device as well as the use of vented animal cabinets.

Despite the absence of a significant reduction in spleen mass, the total cell numbers were reduced in R and HU mice by 20% and 25%, respectively. Our data are in accordance with Wei et al. [35], who observed a decrease of approximately 50% in the splenocyte number from the second day of suspension, mediated by apoptosis and leukocyte redistribution. The same explanations likely apply in our case because the stress response generated *via* the hypothalamic-pituitary-adrenal axis and the sympathetic nervous system (SNS) is known to provoke apoptosis, and it is also known that the head-down tilt position induces a fluid shift. Because our data show that the mice adapted quickly to the R or HU treatments, we can reasonably assume that, in our experiment, fluid shift is the major factor that affects the number of splenic cells. However, we cannot totally exclude the role of the sympathetic nervous system on the spleen, which is abundantly innervated [50]. At the beginning of the suspension, a reduction in the splenocyte number could take place following the activation of the SNS, even in the absence of elevated corticosterone at the end of the suspension, as Wei et al. [35] have suggested.

Our flow cytometry analysis revealed that lymphocyte subpopulations are modified in the spleens of R and HU mice (Fig. 4B), with a greater and more significant effect for the HU group. Thus, the head-down antiorthostatic position adds its effect to that of tail restraint. The proportion of CD19^+^ B cells decreased with a concomitant increase in CD3^+^ T cells in mice exposed to HU, and the B/T cell ratio decreased from 1.2 to 0.5 (C *vs* HU groups). The absolute number of B cells was dramatically decreased (by about 60%) in the spleen of HU mice while the absolute numbers of T cells remained unchanged. Interestingly, the CD4^+^/CD8^+^ ratio decreased from 1.6 to 1.2 (C *vs* HU). Several studies have shown changes in the subpopulations of lymphocytes in the secondary lymphoid organs after exposure to simulated microgravity or after a stay in space. The results were sometimes contradictory and organ-dependent. Sonnenfeld et al. [51] reported an increase in the percentage of splenic CD4^+^ and CD8^+^ cells in rats flown onboard Cosmos 2044 for 22 days, while Pecaut et al. [52] have reported the opposite. Gridley et al. [9] showed a decrease in B cell counts in the spleens of C57Bl/6 mice flown on STS-118, whereas another study [39] revealed the opposite for C57Bl/6 mice flown on STS-108. The discrepancy between these results is somewhat disconcerting. Pecaut et al. [39] have previously discussed these contradictory effects involving not only differences in flight or suspension conditions but also single-label flow cytometric analyses. Here, we used a 5-color flow cytometer to characterize the various phenotypes with a greater degree of accuracy. Moreover, the choice of phenotypic markers can also influence the results and consequently complicate inter-study comparisons. Our data are in agreement with O'Donnell et al. [32], who observed similar specific changes in the lymphocyte subpopulations as early as 18 h after the initiation of the hindlimb unloading procedure using the same markers. Recently, Grigorenko and Sapin [53] have shown a dramatic activation of splenocyte destruction, absence of plasma cells and suppression of lymphocytopoiesis with the disappearance of mitotically dividing cells associated with a decrease in the number of blast cells in Mongolian gerbils after a 12-day-long spaceflight. Overall, the changes were most evident during the first week, with a greater change noted for cells in the spleen. Taken together, these findings indicate that the cell redistribution in the spleen takes place early in response to altered gravity exposure and persists throughout at least three weeks. Two mechanisms are possible to explain this change: a release of B cells from the spleen or a greater sensitivity of B cells to apoptosis. Wei et al. [35] found that HU-induced lymphopenia was due, at least in part, to apoptosis. Nevertheless, B and T cells were impacted in the same proportions. Thus, once again, the redistribution of cells due to changes in fluid distribution associated with the modification of adhesion molecule expression seems more likely. Further studies are needed to determine whether these modifications are associated with changes in adhesion molecule expression and/or in chemokine secretion, as shown after spaceflight [54] and hypergravity exposure [34]. Furthermore, it would be very interesting to determine which type of B cells is affected, e.g. marginal or follicular zones B cells. In any case, our findings demonstrate a shift in immunological homeostasis from the humoral branch of adaptive immunity toward cell-mediated mechanisms, as indicated by the shift from B to T cells in the spleen, especially toward cytotoxic T cells.

Fewer total lymphocytes divided in response to stimulation, regardless of the mitogen, for both the R and HU groups, even if this difference was only statistically significant for the HU group stimulated with LPS (+41.6% *vs* +16.7%, for C *vs* HU groups) (Table 3). Upon analysis of lymphocyte subpopulations, no difference was observed for CD19^+^ cells between the three groups after LPS stimulation. CD3^+^CD8^+^ cells were less increased after ConA stimulation in suspended mice (+139.4% for C *vs +*61.3% for HU). The p value obtained when the C and HU groups were compared was 0.081, likely because of the high inter-individual variability. CD3^+^CD4^+^ cells were not increased in C mice and were even reduced in both the R and HU groups after stimulation (-2% for C *vs* -22% for HU and R). It should be noticed that, in these *in vitro* stimulation studies, we focused on the proportion of the different cell subsets without taking into account absolute cell numbers. Thus, observed variations in percentages of cell subsets cannot exclude the possibility that a loss or an increase of cell survival during the 3 days of culture could impact our interpretation. Nevertheless, our findings are comparable with early studies of rodents exposed to suspension or spaceflight [30], [39], [46], [55]. However, in these studies, the duration of total lymphocyte stimulation was shorter, and the authors used traditional *in vitro* methods such as tritiated thymidine to measure proliferation. These methods provide general information about the broad effects on proliferation, but give no deep insight into how the proliferative response is altered.

To better understand these proliferation results, we calculated the percentages of dividing cells in each sub-population. A dramatic decrease of approximately 40% was noted for CD19^+^ B cells (Fig. 7A). These results clearly demonstrate that the antiorthostatic position has effects beyond those of orthostatic restraint, not only on the proportion of B cells in the spleen but also on their mitogenic response. These decreases in splenic B cell number and proliferative response could greatly impair organism defenses against Gram-negative bacteria, as revealed by the studies of Sonnenfeld's team [28], [56] performed on HU mice infected with *P. aeruginosa* or *K. pneumonia.* Furthermore, the reduced B cell proliferation capacity could have serious consequences for the ability to secrete immunoglobulin.

The percentages of CD3^+^, CD3^+^CD8^+^ and CD3^+^CD4^+^ cells that divided after ConA stimulation were reduced in HU animals (Fig. 7B). Unfortunately, inter-individual variations, which were higher for the response to ConA, resulted in the lack of a significant difference. A close inspection of these perturbations showed that CD3^+^CD4^+^ cells divided in response to ConA (47% *vs* 36% for C and HU groups), even if a diminution of this cell type was observed 72 h after mitogenic stimulation (Table 3). This decrease could be because some stimulated Th cells died before the end of the experiment, as shown by our flow cytometric analyses (Fig. 5A). Decreases in the mitogenic response of T cells caused by altered gravity have been extensively documented [26], [57]–[59]. However, it is important to note that in our study, unlike other experiments [6], [28], [60], cellular changes were not associated with an increase in corticosterone concentration. Our results are consistent with our previous study reporting a reduced lymphoproliferative response after LPS or ConA stimulation in the absence of HPA axis activation for splenocytes of mice centrifuged at 2G for 3 weeks [34]. Many authors were interested in understanding the mechanisms that could explain these changes in T cell activation under altered gravity, either associated or not associated with the stress response. Recently, Chang et al. [61] have revealed that the transcription of early genes was inhibited in human T cells activated *in vitro* during spaceflight. In particular, the transactivation of Rel/NF-κB, CREB, and SRF gene targets were down regulated. Furthermore, Singh et al. [62] have suggested that epigenetic events could be one of the mechanisms for microgravity-induced gene expression changes and associated adverse health effects. Consequently, further research is necessary to investigate the transcription of genes in B cells, particularly those involved in Toll-like Receptor 4 signaling, which, to our knowledge, have never been studied under altered-gravity conditions.

To evaluate the consequences of B-to-T and Th-to-Tc cells shifts on the Th1/Th2 cytokine balance, a secreted cytokine bead array assay was used. No statistically significant difference could be detected in culture supernatants. Only a small increase in IL-6, GM-CSF and IFN-γ could be detected when the splenocytes of HU mice were stimulated with LPS, suggesting an increase in pro-inflammatory cytokine production that was not confirmed after ConA stimulation. These results are in agreement with Nash et al. [63], who found that the ability of mitogen-stimulated lymphocytes to secrete IL-2 and IL-1 was not affected by 7 days of antiorthostatic suspension or after spaceflight. On the other hand, some authors have suggested that the Th1/Th2 balance could be modified in astronauts [64]. However, cytokine regulation appears to be a complex process, subject to subtle physiological perturbations, involving selective alterations of specific cytokine functions [10]. Secretion of cytokines by isolated cells is not subject to the complex *in vivo* physiological environment. In addition, the 72 h incubation time needed to study cell proliferation with CFSE is most likely too long to detect cytokines that are usually secreted during the first hours following stimulation. Further experiments, such as intracellular cytokine quantification, will be required to confirm whether the Th1/Th2 shift exists. If this is the case and the Th2 shift persists during long missions, it could represent a significant clinical risk for Th2-related autoimmune diseases, allergies, hypersensitivity and disease susceptibility related to diminished cell-mediated immunity [65].

In summary, our findings show, for the first time, that antiorthostatic suspension not only reverses the B/T ratio in the spleen but also differentially affects cell-specific mitogenic responses in the absence of increased corticosterone concentration. B cells are more vulnerable to simulated microgravity than T cells, among which Th are more sensitive than Tc. Thus, HU induces a shift in immunological homeostasis from the humoral branch toward cell-mediated mechanisms. This shift may increase the susceptibility to numerous widespread pathogens, such as Gram-negative bacteria, and compromise astronauts' defenses.
